# What’s driving dermatology? Contribution title analysis of the largest German Dermatology Congress 2019

**DOI:** 10.1177/20552076211012138

**Published:** 2021-04-21

**Authors:** Robert Kaczmarczyk, Felix King, Tilo Biedermann, Alexander Zink

**Affiliations:** Department of Dermatology and Allergy, School of Medicine, Technical University of Munich, Munich, Germany

**Keywords:** Digital health, digital, health informatics, epidemiology, network analysis, personalised medicine

## Abstract

**Background:**

Every two years, German-speaking dermatologic specialist groups gather in Berlin to share the latest developments at Germanýs largest dermatologic conference, the Annual Meeting of the Germany Society of Dermatology (DDG). Because this conference has a lasting effect on dermatologic practice and research, understanding what is moving the specialist groups means understanding what is driving dermatology in Germany.

**Methods:**

We used word network analysis to compile and visualize the information embedded in the contribution titles to the DDG Annual Meeting in 2019. We extracted words, contributing cities and inter-connections. The data was standardized, visualized using network graphs and analyzed using common network analysis parameters.

**Results:**

A total of 5509 words were extracted from 1150 contribution titles. The most frequently used words were “therapy”, “patients”, and “psoriasis”. The highest number of contributions came from Hamburg, Berlin and Munich. High diversity in research topics was found, as well as a well-connected research network.

**Conclusions:**

Focus of the well-connected German-speaking dermatology community meeting 2019 was patient and therapy centered and lies especially on the diseases psoriasis and melanoma. Network graph analysis can provide helpful insights and help planning future congresses. It can facilitate the choice which contributors to include as imbalances become apparent. Moreover, it can help distributing the topics more evenly across the whole dermatologic spectrum.

## Introduction

Dermatology is a broad medical field with a vast scientific ecosystem.^1^ Especially in Germany, Dermatology with its long tradition is a broad medical field including many different subspecialities such as – among others – allergology, dermatooncology, dermatologic surgery, dermatopathology, aesthetic dermatology, andrology and microbiology.^2^ In the last few years, dermatology has experienced a great thrust forward as therapies were developed based on new insights into the pathogeneses of several dermatologic diseases operating on a molecular level.^3–7^ It is also a time of change, where old truths become irrelevant and less goals seem out of reach.^8^ Today, for example patients with diseases like psoriasis, once considered untreatable, have a real chance of being symptom-free and living a normal life.^8^

Once every two years, the German-speaking community together with a selection of clinicians and scientists from all over the world convenes at the Meeting of the German Society of Dermtology (DDG) in Berlin. At this meeting current research as well as new clinical findings are shared and discussed in lectures, symposia, smaller groups, workshops and posters. Lessons learned at the meeting from lectures and medical training courses at the conference can be translated into dermatologic practice.

Visualization through network graphs can enable humans and especially scientists to understand intricate, multi-dimensional and closely intermeshed data. It is already used widely within the digital humanities including for example social^9^ and political sciences.^10^ Medical sciences have yet to discover the full potential of networkanalysis, which seems especially powerful in understanding and verifying the output of increasingly published techniques and systems using artificial intelligence.^11,12^ Analyzing keywords has been done scarcely across the medical field, an exception being epidemiology^13^ and public health.^14^ However, it is a very effective way to reveal networks and trends in large samples of text-based data. Because the bienial DDG meeting is so influential within the German-speaking dermatology community, the goal of this work was to assess the relevant topics and to highlight overall trends and movements in order to reflect what’s currently driving dermatology and in which direction modern dermatology is moving.

## Material and methods

### Study design

We performed a retrospective analysis of all contribution titles in the program of the 50th anniversary congress of the DDG which took place in Berlin, Germany from the 1st to the 4th of May 2019 with 3098 participants. The contributions could be submitted between the 2nd of July to the 11th of November 2018. Late-breaking submissions were accepted between the 1st of December 2018 till the 6th of January 2019.

### Setting

All congress contributions can be classified into 9 categories: courses, working groups, free lectures, plenary lectures, keynote lectures, clinical slideshows, symposia, industry lectures and posters. Contributions can be further divided into open (posters and free lectures submitted within the deadlines) and compulsory (courses, working groups, plenary lectures, keynote lectures, clinical slideshows, symposia and industry lectures) presentations.

### Variables

The category, cities and titles were analyzed for all program contributions. The titles were divided into separate word and word groups for further analysis.

### Data sources/measurement

All data were taken from the official conference program pdf file.^15^ Data were extracted from the pdf file using tabula v1.2.1 open-source software. Filling words like ‘and’ were excluded from the analysis, as well as duplicate titles and words to prevent an artificial increase in word counts.

### Software

Python’s project ‘langdetect v1.0.7’, a direct port of Google’s language detection library, was used to identify the English titles and ‘googletrans v2.4.0’, a Google translation API, to translate the English titles into German. After translation, the titles were standardized semi-automatically using custom-written python code (e.g. plurals and singular forms, synonyms). Disease entities with names with more than one word were split into fragments (e.g. atopic dermatitis into atopic and dermatitis). This was necessary to get a more reliable output because multiword terms, often spelled aberrantly, can decrease the software performance. The cities to which the authors were affiliated were separated from the title analysis.

The network was drawn using a ForceAtlas 2 layout.^16^ Nodes and their titles were sized proportionally to their counts. In the titles network graph, the node degree cutoff was 20, whereas in the city network all nodes and connections were drawn. Labels were drawn for all visible nodes. The parameters were manually adjusted to guarantee a comprehensive overview of each network (Tolerance: 0.1, Approximation 1.0, Scaling: 10, Stronger gravity, Gravity: 0.1, Dissuade Hubs, LinLog mode, Prevent Overlap, Edge Weight Influence, 1.0 – export parameters: Border Width 1.0, Border Color: parent, Opacity: 100, Show Labels, Font Arial 12 Plain, Proportional node size, Max characters: 30, Outline opacity: 80, Edge Thickness: 10, Min. rescaled weight: 0.01, Max. rescaled weight: 10.0, Opacity: 50, curved radius).

### Quantitative variables

We analyzed typical network variables calculated by Gephi v.0.9.2^17^ open-source software to examine the contribution and city networks. A network consists of nodes and edges. A node describes a single word/word group or city, and an edge a connection between two nodes. All word-groups or cities from one submission are connected by edges. The same nodes from different contributions appear within the same single node in our network.

The degree of a node describes the number of edges from that specific node, and the average degree the average of all nodes’ degrees. The average path length is the average distance between the nodes of a network, wherein connected nodes have a graph distance of 1. Network density examines how close a network is from a complete network, where every node is connected to every other node. A complete network’s density equals to 1. Modularity measures the strength of division of a network into compartments. A network with higher modularity has dense connections within the compartments and sparse connections between the compartments. Different colors within a network represent different modularity classes.

In the context of our analysis, the degree of a word or city, the ‘node’, describes the number of connections, the ‘edges’, to and from that specific word or city. Every single node has connections to every other node within a single title. The same nodes from different titles appear in the same single node in our network graphs.

### Statistical methods

Statistical analysis was carried out using Microsoft Excel, Python’s ‘pandas v0.24.2’^18^ and ‘collections’ library and Gephi v0.9.2^17^ open-source software. Since we extracted the submissions from the pdf file of the official conference program,^15^ we did not have missing data (except for removed duplicates and fill words, which were not included in the analysis). We created two network graphs, one for all contribution titles and the other for contributing cities. If more than one author of one contribution was from the same city, that was only counted as one contribution from the respective city in order not to heighten the count artificially.

We presented the title word counts for each skin disease proportionally to all listed skin diseases. The numbers are either absolute counts, percentages or both.

## Results

**Figure 1.**
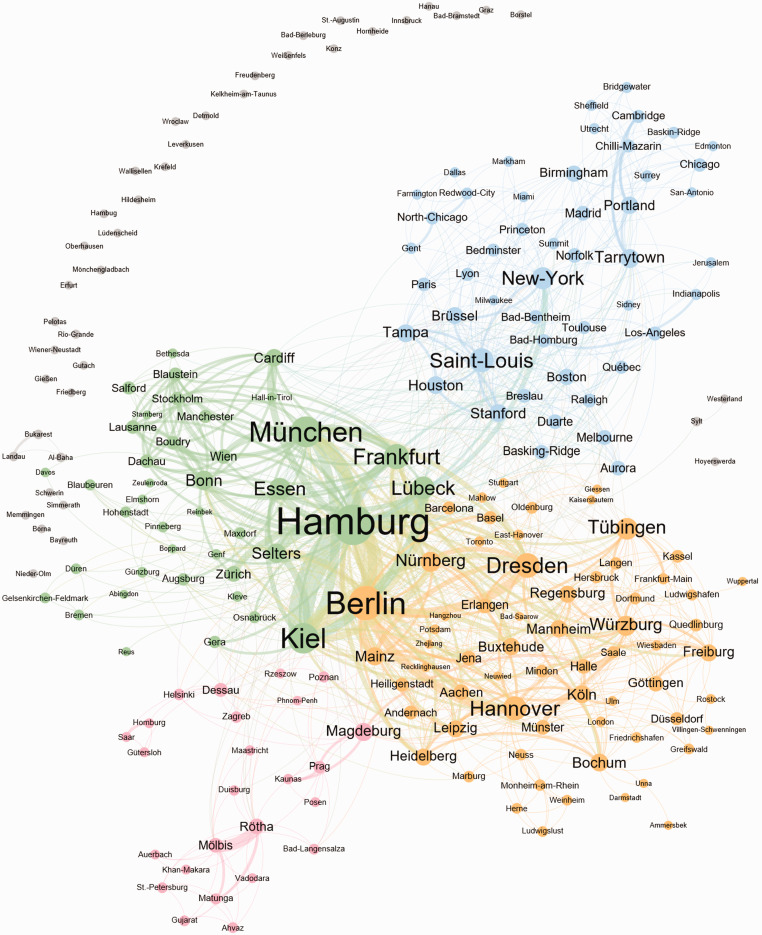
Cities network graph with separated communities based on modularity levels of contributions to the congress of the DDG 2019. This network graph shows all 223 cities (nodes) and 1083 city connections (edges) of all contributions of the 50th congress of the German dermatologic society in Berlin 2019. The 4 colors (red, blue, green and yellow) represent one community each within the network graph. Grey subsumes all other 42 communities. Based on the modularity level, a community groups cities which appear together more often and thus have more connections between them.

All in all, we extracted 5509 words or word groups and 1596 cities from 1150 titles of the program of the 50th Meeting of the Germany Society of Dermatology (DDG) in April 2019 in Berlin, Germany. In total, sixty-eight of the 1150 titles (5.9%) were in English language, which were translated into German using Google’s translate API. Naturally and es expected for a German-speaking meeting, the majority of contributions came from Germany, followed by other European countries and the Unites States of America with a total of 223 different contributing cities. Some contributions even came from as far as Pelotas, Brazil and Phnom-Penh, Cambodia (Figure 1).

**Figure 2.**
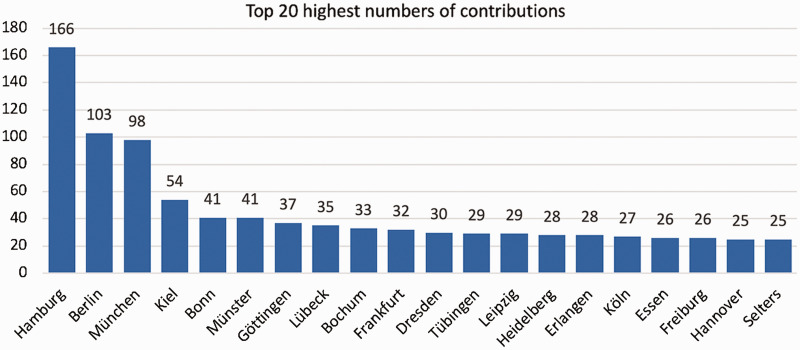
Top 20 most contributing cities to the congress of the DDG 2019. This figure shows the number of total contributions of cities sorted in descending order. The three largest cities in Germany, Hamburg, Berlin and Munich already account for 22,99% of all contributions made to the congress.

**Figure 3.**
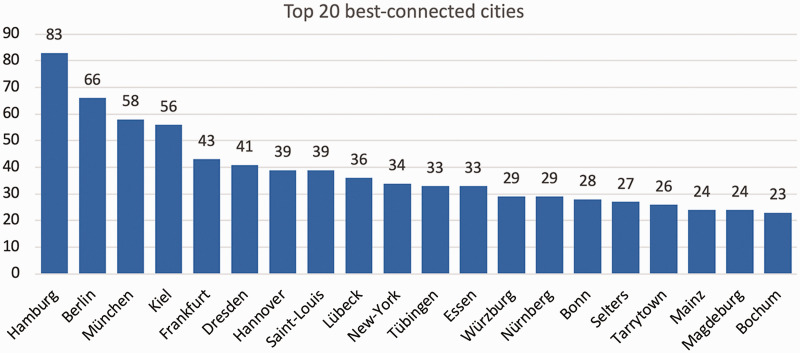
Top 20 best-connected cities to the congress of the DDG 2019. This figure shows the number of connections, the degree, of each city contributing to the congress. Again, Hamburg, Berlin and Munich rank among the three best connected cities.

The 10 cities with the most contributions were Hamburg (n = 166), Berlin (n = 103) and Munich (n = 98), representing the three largest German cities by inhabitants, followed by Kiel (n = 54), Münster (n = 41), Bonn (n = 41), Göttingen (n = 37), Lübeck (n = 35), Bochum (n = 33) and Frankfurt (n = 32) (Figure 2). Apart from cities in Germany, the highest number of contributions were counted for Zurich, Switzerland (n = 12), Vienna, Austria (n = 9), New-York, United States (n = 7), Basel, Switzerland (n = 7), Prague, Czech Republic (n = 4) and Cardiff, United Kingdom (n = 4). The Top 10 contributing cities in terms of collaboration with other cities were Hamburg with 83 connections to other cities, Berlin (n = 66), Munich (n = 58), Kiel (n = 56), Frankfurt (n = 43), Dresden (n = 41) and Hannover (n = 39) (all Germany), Saint-Louis (USA) (n = 39), Lübeck (GER) (n = 36) and New-York (USA) (n = 34) (Figure 3). The city network graph reveals four big communities, wherein cities are more likely to work together than between different communities: Two German communities, one British-American community led by Saint-Louis (n = 39), New-York (n = 34) and Tarrytown (n = 26) and a Scandinavian-Eastern-European community. These four represent the biggest communities at the DDG conference 2019 with all other 95 communities being relatively small in comparison (Figure 1). These other 95 communities are mainly cities with few or no connections and were mainly part of the ‘courses’-section. On average, one city was connected to 10 other cities and 4.4% of all possible city connections were observed (network density). A network density of 100% means, in the context of collaborations, that every city collaborates with every other city at least on one project. A city can reach any other city on average within 2.6 connections (average path length: 2.6). This shows a well-connected dermatologic research community.

**Figure 4.**
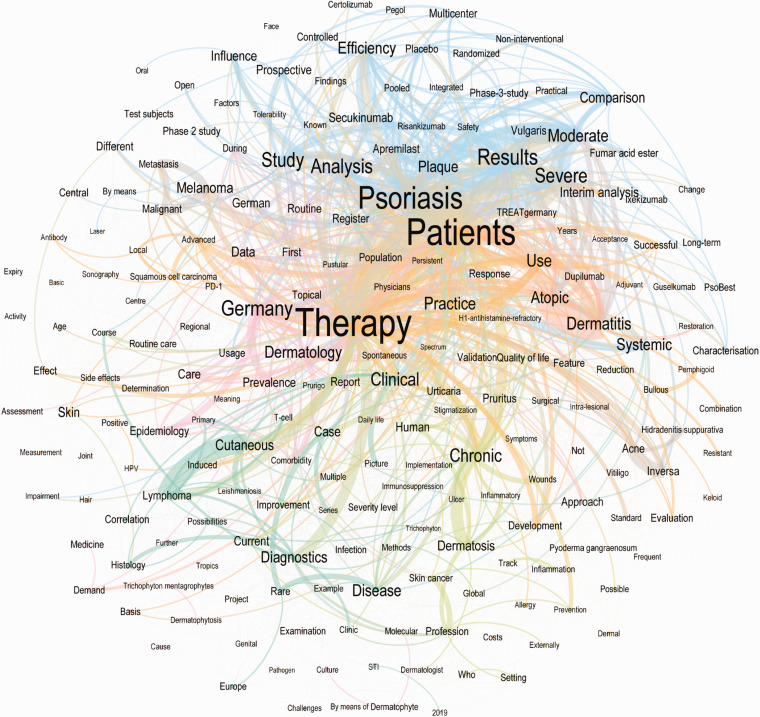
This network graph shows words (nodes) and word connections (edges) of all contributions of the 50th congress of the German dermatologic society in Berlin 2019. Only 209 words (10% of all analyzed words) which have at least 20 connections and 2357 word-connections (20.15% of all connections) are shown. The colors represent one community each within the network graph. A community groups words together which appear together more often and thus have more word connections between them. Note that separate terms, which one might assume could be an issue, appear together in a graph (e.g. dermatitis and atopic or cutaneous and lymphoma).

**Figure 5.**
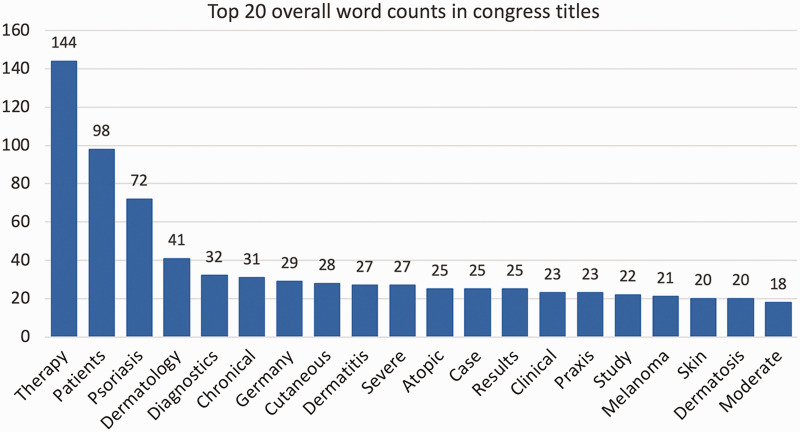
Top 20 overall word counts in congress titles. This figure shows the word ranking in descending order from all contributions at the 50th DDG congress in Berlin 2019.

After translating the German titles into English, the word with the highest overall counts was “therapy” (n = 144), followed by “patients” (n = 98), “psoriasis” (n = 72), “dermatology” (n = 41), “diagnostics” (n = 32), “chronical” (n = 31), “Germany” (n = 29),” cutaneous” (n = 28), “dermatitis” (n = 27) and “severe” (n = 27) (Figure 4, 5). The poster contributions were further divided into 36 subcategories, in descending order “Care research” (n = 49), “educative case (therapy)” (n = 31), “educative case (diagnostics)” (n = 30), “clinical studies” (n = 27), “pediatric dermatology” (n = 26) and “microbiology” (n = 21).

**Figure 6.**
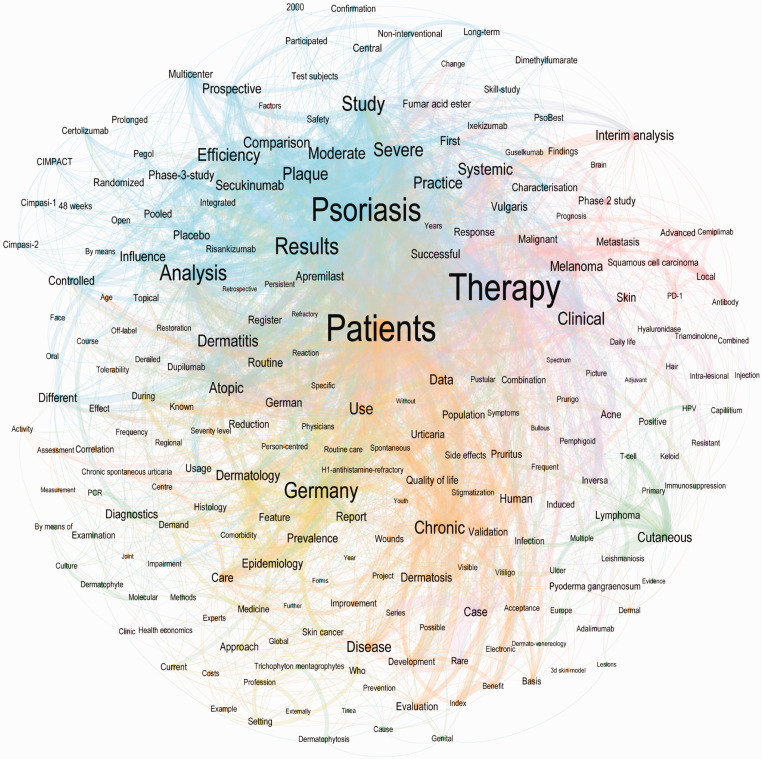
The network graph of the open submission titles of the congress of the DDG 2019. These network graphs show a subset of all nodes (words) and word connections (edges) of the congress contributions’ titles, separated into open submissions. Only nodes with at least 16 connections are shown for the open network graph, resulting in 228 visible nodes out of 1443 and 2428 visible edges out of 9157. In the compulsory network graph, only nodes with at least 7 connections are shown, resulting in 258 visible nodes out of 903 and 1211 visible edges out of 2705. The different colors represent word groups with the same modularity class.

**Figure 7.**
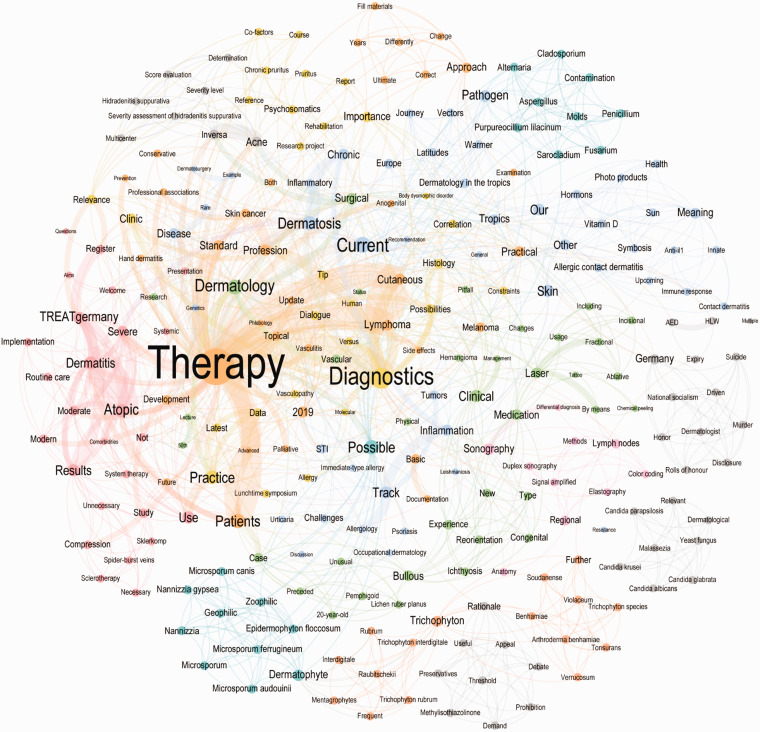
The network graph of the compulsory submission titles of the congress of the DDG 2019. These network graphs show a subset of all nodes (words) and word connections (edges) of the congress contributions’ titles, separated into and compulsory contributions. Only nodes with at least 16 connections are shown for the open network graph, resulting in 228 visible nodes out of 1443 and 2428 visible edges out of 9157. In the compulsory network graph, only nodes with at least 7 connections are shown, resulting in 258 visible nodes out of 903 and 1211 visible edges out of 2705. The different colors represent word groups with the same modularity class.

**Figure 8.**
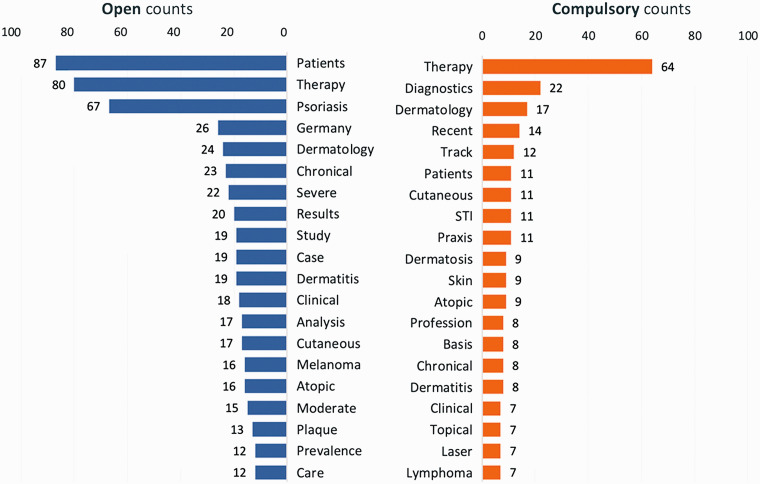
Top 20 word counts in open (left) and compulsory (right) submissions titles. The graph shows a side by side comparison of total word counts in open vs compulsory contributions’ titles. The numbers represent absolute values.

The subgroup analysis of the network graph reveals differences and similarities between open and compulsory contributions (Figurs 6-8). The top 10 highest word counts in the open submissions were seen for “patients” (n = 87), “therapy” (n = 80), “psoriasis” (n = 67), “Germany” (n = 26), “dermatology” (n = 24), “chronical” (n = 23), “severe” (n = 22), “results” (n = 20), “study” (n = 19) and “case” (n = 19), whereas the highest word counts for compulsory contributions were “therapy” (n = 64), “diagnostics” (n = 22), “dermatology” (n = 17), “recent” (n = 14), “track” (n = 12), “patients” (n = 11), “cutaneous” (n = 11), “STI” (n = 11), “practice” (n = 11) and “dermatosis” (n = 9) (Figures 6–8).

A focus on therapy and clinical practice can be derived from the analysis, both open and compulsory contributions. The title network parameters revealed that on average, 1 word was connected to 11 other words and 0.5% of all possible word connections were detected. The average path length is 3.2, which means that the average distance between any two words is 3.2 connections. The network parameters for open and compulsory contributions show slight differences: The compulsory network graph has a higher network diameter and average path length but a lower graph density and average degree.

## Discussion

Our network analysis of the 2019 conference meeting of the Germany Society of Dermatology (DDG) reveals a well-connected German-speaking Dermatology-community stretching across Germany, Europe and beyond. The most prolific contributors 2019 were Hamburg, Berlin and Munich with an overall strong focus on patients and therapy. The skin diseases that gained the most attention were psoriasis, atopic dermatitis and melanoma.

This analysis of the contribution titles using word network analysis visualizes unprecedented insights into the inner linings of the dermatologic community. Its low network density indicates a high diversity in dermatologic research. An explanation for sure is the ongoing development of several new therapeutic options^19^ especially targeting patients with chronic inflammatory skin diseases (e.g. psoriasis^20^) and skin malignancies (e.g. melanoma^21^) using antibody medications. This is supported by the fact that psoriasis, atopic dermatitis and melanoma were the skin diseases most assessed by the contributions. Another focus was the teaching cases for clinical practice which highlight the educational aspect of the 2019 DDG meeting.

The comparison of open and compulsory contributions network parameters reveals a higher diversity of the compulsory contributions – this could be explained by the fact that there were more categories in the compulsory group.

Analyzing the DDG 2019 contribution titles using word network analysis provides an account of topics and co-operation networks. The data is visualized in comprehensive graphs and can be examined by professionals of all disciplines as well as even laypersons without further data science training. A detailed analysis of all subcategories might be able to reveal their different focus of interest in the future. Longitudinal analyses of contributions could delineate trends and derive predictions. Apart from that, funding sources of the contributions, quality of life data, overall PubMed results on different dermatologic topics as wells as disability-adjusted life years from skin diseases could be compared to our network parameters, which might give a broader and more complete account on this topic.

A downfall of the method is a difficult and time-consuming process of standardization. A researcher must list all possible semantic expressions and register synonyms and word groups. Qualitative categorization is difficult when a title’s hints at certain pathogens which cause skin and other diseases without naming the exact disease. Another limitation is the analysis of a German congress which, although with international participation, shows mostly German research interests.

Of course, not every specialist within the German-speaking dermatology community contributed to the DDG 2019. Evaluating the DDG 2019 contribution titles therefore can only offer a snapshot of the research community. Also, there is a publication bias, as some authors contributed a lot while others could have contributed their work but for whichever reason chose not to do so. Another limitation is the evaluation only of the title of the contributions, not the complete abstracts where available, which could have influenced our findings in either ways, over- or underrepresenting different topics. Most important, however, it has to be highlighted that network analysis can only assess the quantity but not the quality of the contributions. In this study, we did not differentiate between the different types of studies that were submitted (e.g. case report vs. randomized clinical trial) nor does the analysis take into account the actual proportion of the work among the different authors of a multicity contribution.

As word network analysis of contribution titles offers valuable insights and easy to understand visualizations of complex context, they seem very useful for a variety of different application areas. This tool could be used to overview and summarize but also meticulously study medical conferences of all specialities including non-medical conferences by analyzing their overall contributions as well as different partial aspects potentially of interest such as for example the number and connection of different drugs described in psoriasis in different contributing cities and countries. Moreover, planning future congresses with the help of network analysis could result in a more balanced selection of diseases, topics and contributing cities as well as the chance to pool different contributors by overall interest and their network.

Through a further standardization of the preprocessing of a submission, mistakes would be reduced and the word network analysis would become more accurate and easier to fashion. Requiring to provide keywords for every contribution by the submitter could be a quick and easy to implement way towards this direction.

The focus of a well-connected dermatology community contributing to DDG 2019 lies on therapeutic approaches to chronic inflammatory skin diseases, namely psoriasis and atopic dermatitis, and melanoma. It is likely that this trend is associated with the now increased insight into the pathogenesis in these fields.^22^ Future research should include longitudinal network analyses to delineate trends; more detailed subcategory analyses will help to differentiate between the focus of interest of the various contributors – for now, only the open to the compulsory contributions’ titles are compared.

Network analysis is very powerful because it can use complex multi-dimensionals sets of data and provide compilations and graphs. It helps in understanding the focus of dermatologic congresses and further detects imbalances of the topics and the selection of the contributors. At the same time, the visualiziation are easy to understand even for people without further data science training. This makes this tool potentially very useful in the assessment and (human) evaluation of results provided by artificial intelligence tools or maching learning, which will for sure change and enrichen medicine, and dermatology.

## References

[1] Ring J. Progress in dermatology and venereology – editor’s pick of the year 2018. J Eur Acad Dermatol Venereol JEADV 2019; 33: 7–10. 10.1111/jdv.15394 30656764

[2] Hensen P Müller ML Stadler R, et al. Dermatologic subspecialties in German inpatient dermatology: a national survey. J Dtsch Dermatol Ges 2008; 6: 735–740. 18371050 10.1111/j.1610-0387.2008.06677.x

[3] Herberger K Dissemond J Brüggestrat S, et al. Biologika und immunglobuline für die therapie des pyoderma gangraenosum – Analyse von 52 patienten. J Dtsch Dermatol Ges 2019; 17: 32–42. 10.1111/ddg.13741_g 30615279

[4] Haiges D Fischer J Hörer S, et al. Biologika-Therapie mit anti-IL-17A-Antikörper verbessert kongenitale ichthyosiforme verhornungsstörung. J Dtsch Dermatol Ges J Ges 2019; 17: 70–72. 10.1111/ddg.13716_g 30615287

[5] Lehmann J Seebode C Emmert S. Research on genodermatoses using novel genome-editing tools. J Dtsch Dermatol Ges J Ges 2017; 15: 783–789. 10.1111/ddg.13270 28622433

[6] Bleuel R Eberlein B. Therapeutic management of vitiligo. J Dtsch Dermatol Ges J Ges 2018; 16: 1309–1313. 10.1111/ddg.13680 30335222

[7] Fathi R Armstrong AW. The role of biologic therapies in dermatology. Med Clin North Am 2015; 99: 1183–1194. 26476247 10.1016/j.mcna.2015.07.008

[8] Rønholt K Iversen L. Old and new biological therapies for psoriasis. Int J Mol Sci 2017; 18: 2297. 10.3390/ijms18112297 PMC5713267 29104241

[9] Borgatti SP Mehra A Brass DJ, et al. Network analysis in the social sciences. Science 2009; 323: 892–895. 19213908 10.1126/science.1165821

[10] Ward MD Stovel K Sacks A. Network analysis and political science. Annu Rev Polit Sci 2011; 14: 245–264.

[11] Yeung S Downing NL Fei-Fei L, et al. Bedside computer vision – moving artificial intelligence from driver assistance to patient safety. N Engl J Med 2018; 378: 1271–1273. 29617592 10.1056/NEJMp1716891

[12] Rajkomar A Dean J Kohane I. Machine learning in medicine. N Engl J Med 2019; 380: 1347–1358. 30943338 10.1056/NEJMra1814259

[13] Peter RS Brehme T Völzke H, et al. Epidemiologic research topics in Germany: a keyword network analysis of 2014 DGEpi conference presentations. Eur J Epidemiol 2016; 31: 635–638. 26994764 10.1007/s10654-016-0141-y

[14] Luke DA Harris JK. Network analysis in public health: history, methods, and applications. Annu Rev Public Health 2007; 28: 69–93. 17222078 10.1146/annurev.publhealth.28.021406.144132

[15] Deutsche Dermatologische Gesellschaft, Vereinigung Deutschsprachiger Dermatologen e.V. 50. DDG-Tagung Citycube Berlin 1. – 4. Mai 2019 Hauptprogramm, https://derma.de/ddg-tagungen/50-ddg-tagung/ (accessed 1 July 2019).

[16] Jacomy M Venturini T Heymann S, et al. ForceAtlas2, a continuous graph layout algorithm for handy network visualization designed for the Gephi software. PLoS One 2014; 9: e98679. 24914678 10.1371/journal.pone.0098679 PMC4051631

[17] Bastian M Heymann S. Gephi: an open source software for exploring and manipulating networks. Qual Saf Health Care 2009; 13: 361–362.

[18] McKinney W. Data structures for statistical computing in python. In: *9th PYTHON IN SCIENCE CONF. SciPy 2010* (eds S van der Walt and J Millman), Austin TX, June 28–July 3 2010, pp.51–56.

[19] Erichsen CY Jensen P Kofoed K. Biologic therapies targeting the IL-23/IL-17 immune axis for the treatment of moderate-to-severe plaque psoriasis: a systematic review and meta-analysis. J Eur Acad Dermatol Venereol JEADV 2020; 34: 30–38. 10.1111/jdv.15879 31419343

[20] Roenneberg S Biedermann T. Pityriasis rubra pilaris: algorithms for diagnosis and treatment. J Eur Acad Dermatol Venereol JEADV 2018; 32: 889–898. 29247481 10.1111/jdv.14761

[21] Zacher M Heppt M Berking C. Triple-Therapie bei metastasiertem melanom. Hautarzt 2018; 69: 25–27. Oct 30264300 10.1007/s00105-018-4214-6

[22] Assaf M Salah E. Lesional upregulation of SLURP1 immunostaining parallels disease severity in psoriasis vulgaris patients. J Dtsch Dermatol Ges 2018; 16: 1329–1337. 10.1111/ddg.13682 30395407

